# Er^3+^: Yb^3+^ ions embedded ZnO nanostructure for photonic applications

**DOI:** 10.1038/s41598-025-24424-7

**Published:** 2025-11-12

**Authors:** H. A. El Meleegi, M. E. Sayed, L. I. Soliman, D. Atta, I. K. Battisha

**Affiliations:** 1 Electron Microscope and Thin Film Physics Department, Physics Research Institute, National Research Centre (NRC), 33 El Behooth St., Dokki, Giza, 6001461 Egypt; 2 Basic Science Department, Modern Academy for Engineering and Technology in Maadi, Cairo, Egypt; 3 Solid State Physics Department, Physics Research Institute, National Research Centre (NRC), 33 El Behooth St., Dokki, Giza, 6001461 Egypt; 4 Spectroscopy Department, Physics Research Institute, National Research Centre (NRC), Dokki, Giza, 6001461 Egypt; 5 Nonlinear Optical Properties and Fluorescence Unit, Physics Research Institute, National Research Centre (NRC), Dokki, Giza, 6001461 Egypt; 6 Electric and Dielectric Materials Measurement Unit, Physics Research Institute, National Research Centre (NRC), 33 El Behooth St., Dokki, Giza, 6001461 Egypt

**Keywords:** Nano-structured, Sol–gel, ZnO thin film, Raman spectroscopy, Up and down conversion fluorescence, And solar cell, Applied physics, Materials for devices

## Abstract

Thin films of nano-structure pure ZnO and co-doped with 0.5 mol% Er^3+^, and different concentrations (0.5, 1, and 1.5 mol%) of Yb^3+^ ions, coded as S, S1, S2, and S3, respectively, have been prepared. Afterwards, the prepared samples were sintered at 500 °C for three hours in air. ZnO films have a wide direct band gap of 3.37 eV and a single-phase hexagonal wurtzite polycrystalline structure. They were prepared using the seed solution spin coating sol–gel method (SSSCSGM). By XRD analysis, the presence of the nano-structured phase was revealed. Over a broad spectrum, including the visible region, optical transparency is demonstrated by spectrophotometry. The produced samples have been characterized using different spectroscopic techniques, including Raman confocal microscope (RCM) and photoluminescence (PL). The latter was used to detect the PL in both directions (up and down-conversion) utilizing a 635 nm excitation laser source. It was observed that the prepared samples exhibited green up-conversion emission at 350 and 493 nm, as well as red down-conversion emission at 1274 nm, due to the efficient energy transfer process between Er^3+^ and Yb^3+^ ions. It is proven that (S3) had the highest up-conversion from the edge of the NIR to the middle of the visible region, due to a spectral overlap energy transfer between the ^2^F_5/2_ band of Yb^3+^ and the ^4^I_11/2_ band of Er^3+^ ions. After depositing the S3 sample at the front of silicon solar cell-based devices, the efficiency of collecting solar cell energy increased during the experiment, rising from 10.5% (before S3 deposition) to 13.1% (after S3 deposition), respectively. Consequently, the S3 film offers the advantage of being extremely easy and reasonably priced to manufacture, making it a viable option for Si-solar cells as an energy-conversion layer.

## Introduction

Photovoltaic (PV) devices can easily convert the free, abundant, and clean energy source (sunlight) into electric energy. The majority of PV cells exhibit inherent insensitivity to the entire solar spectrum, and their response is limited to a small range above the bandgap (Eg) of the light-absorbing material^1^. Theoretically, p-n junction solar cells can only have a maximum conversion efficiency of 30%^2^, with the majority of energy loss coming from a mismatch in wavelengths between the sun’s light spectrum, and the light-absorbing material’s Eg. PV cells do not absorb incident photons with energies less than Eg; this loss typically happens in a PV cell with a wide Eg. However, once more, a significant portion of the surplus energy is released as thermal energy^3,4^. On the other hand, photons with an energy greater than Eg are absorbed and converted to electric energy. Therefore, there are two ways to improve the conversion of sunlight into electric energy: either choose appropriate light-absorbing materials to maximise cell performance, or manage incident photons so that they better match the absorption wavelength range of the solar cell. UV photons with higher energy can be converted to visible or near-infrared (NIR) photons, which can be efficiently absorbed by the solar cell, by putting down-conversion (DC) and/or down-shifting (DS) layers in front of the cell^1,4,5^. Still, unfortunately, these layers are unable to do the same for the NIR region of the sun’s spectrum. PV cells with a wide Eg can also be made more efficient by using up-conversion (UC) layers, which double the frequency of low-energy photons by energy transfer to form a single photon with an energy greater than the Eg^1^..

Because lanthanide group elements have available energy levels for effective spectral conversion, they are commonly employed for both UC and DC/DS materials. Effective photon management using one or two types of lanthanide doping has been reported in several recent investigations^6–8^. The remarkable properties of nanostructured wurtzite zinc oxide thin films and related compounds have piqued the interest of scientists. These include high electron mobility, high absorption and dispersion of UV radiation, a broad band gap energy (E_g_) of approximately 3.4 eV, and strong binding energy for excitons of about 0.06 meV at room temperature, non-toxicity, low cost, optical transparency, high resistance, piezoelectric capabilities, and other features^9,10^. It has extensive applications in biophysics, photoluminescence, optoelectronics, gas sensing, piezoelectric materials, and catalysis^11–13^. The Er^3+^ and Yb^3+^ ions present a spectral overlap between the ^2^F_5/2_→^2^F_7/2_ band of Yb^3+^ and the ^4^I_15/2_→^4^I_11/2_ band of Er^3+^, allowing the transfer of energy between these two rare earth ions, which enhances the photoluminescence up-down conversion (PLUDC) process^14–16^. Numerous methods, including vapor deposition, precipitation in aqueous solution, hydrothermal synthesis, mechanochemical processes, and the sol-gel process, which is employed in the present work, are used to create various nanostructured thin films and powder materials due to their adaptability and well-planned mass production capabilities^17–19^. Molecular spectroscopy helps identify the chemical structure of a vast spectrum of samples, ranging from archaeological samples^20–22^, biological samples^23,24^, and biochemical substances^25–30^ to materials science and solid-state samples^31–33^. Moreover, atomic spectroscopy is regarded as an essential tool to recognize the elemental structure, such as atomic absorption, XRF, neutron activation, XPS, or even the crystal structure^34,35^. One of the recently researched areas is modeling-assisted molecular spectroscopy, which confirms vibrational modes and optical transitions with very high accuracy^36–39^. On the other hand, optical imaging and micrographs can provide valuable information about the surface of synthesized samples, including those synthesized of metal^40–43^.

The present work aims to study the Up and down conversion Fluorescence methods for co-doped nano-structure ZnO with Er^3+^ activated by Yb^3+^ ions thin films, to be investigated as a potential means to transform infrared (IR) photons, which have an energy level below that of solar cells, into visible light, which has an energy level greater than that of solar cells.

## Experimental

### Materials and methodology

Nano-structured Wurtzite hexagonal pure ZnO thin film doped with 0.5 mol. % of Er^3+^ ions, and different concentration of Yb^3+^ ions at 0.5, 1 and 1.5 mol. %, assigned to S1, S2, and S3, were fabricated using SSSCSGM and deposited on a precleared glass substrate. For the best quality and surface morphology of the synthesized films, the substrates were cleaned before the deposition of the nano-crystal ZnO thin film using a sonic cleaner in ethanol for 15 min, as previously illustrated^10^. This process helps remove dust and unwanted chemicals from the substrate surface, providing a clean surface that can be used for further procedures. A coating solution was prepared by dissolving zinc acetate dehydrate (ZnCH_3_COO)_2_ 2H_2_O) in mix solution mono-ethanolamine (NH_2_CH_2_CH_2_OH) (MEA) and 2-methoxyethanol at room temperature, however the molar ratio of MEA to zinc acetate was 1:1. The seed solution was stirred at 50 °C for two hours until yielding a clear and homogeneous solution (Solution 1). Then, the Er^3+^ ions were introduced in the process by mixing Erbium Nitrate solution with molar ratios of 0.5–1.5 mol% of Er_2_O_3_ with (Solution 1) to obtain (Solution 2). The resulting homogeneous Solution 2 was dropped onto the glass substrate, which was rotated at 4000 rpm for 30 s, by using a spin coater. The coating procedure was repeated multiple times, followed by air-drying at ambient temperature, and then subjected to a preheated laboratory oven at 150 °C. In order to achieve the desired outcome of ZnO nanostructure films, this was done with the intention of accelerating the dehydration of zinc acetate onto the glass substrate. The films subjected to a thermal treatment chamber that was heated to 500 degrees Celsius for a period of 180 min.

### Analysis

#### XRD

Experimental XRD crystallographic analysis has been carried out on the thin films. X-ray diffractometers, which made by Malvern Panalytical (which was originally known as Philips), were used to ascertain the degree of crystallinity present in these compounds. λ= 1.5418 Å, and the device operated on monochromatic CuKα1 radiation at 0.40 mega Volt and 0.3 A. To determine the size of the crystallite (β), we use the following Scherrer’s equation:

1 $$\beta = \frac{K\lambda }{{D Cos\left( \vartheta \right)}}$$

The variables K, λ, and D are pointed out as Scherrer’s constant, wavelength, and FWHM of the pattern, respectively, and measured in radians. An adjustment made to the FWHM of the recorded XRD band (Ds) to consider the systematic widening generated by the instrument. The diffraction scan of a strain-free powdered quartz sample provided the peak widths (Dq), which used to make this correction. The diffraction scan conducted on a quartz specimen with crystallite sizes ranging from five to ten microns using the same parameters. D = (Ds^2^−Dq^2^)^1/2^ was used to calculate the computed adjusted sample peak widths. The Win-Fit tool used to analyze the micro-strain and crystallite size impacts of D. The expansion of the instrument measured using standard samples.

#### Optical measurements

A Japanese JASCO V-570 double-beam spectrophotometer used for the measurement process to determine the transmittance and reflectance spectra. With a wavelength range that extended from 0.2 to 2.500 µm, the spectrophotometer had an accuracy of around 0.0022%.

#### Optical imaging

An optical microscope, specifically an inverted laser scanning microscope from Olympus (IX-83), has been employed in bright-field imaging mode. All the data were photographed using a CCD camera. For the scanned area of each sample, a fixed size of 600 × 500 µm was selected.

#### Raman confocal microscope

German manufacturer Witec’s confocal Raman microscope is armed with a 20X objective lens made by Ziss and a 532 nm picosecond pulsed laser.

The film surface remained unaffected, and the samples were not heated, resulting in a widening of the Raman spectra once the laser power was optimized. In contrast, the more powerful indicates that most Raman signals are captured. During the 20-frame Raman point measurements, the laser power was calibrated to 5 mW. We used a grating with a groove density of 1200 grooves/mm.

#### Fluorescence spectroscopy

The FS5 spectrofluorometer, manufactured in Edinburgh, United Kingdom, was used to perform the PL measurements. To conduct studies involving up-down conversion, 635-nm laser sources were used.

#### Covering the solar cell

For the purpose of examining the impact of applying the developed sol–gel samples on the efficacy of the solar cell, a layer of ZnO, which has been loaded with Er^3+^ and Yb^3+^ ions, has been coated onto the solar cell’s interface. According to the information presented in Section “Materials and methodology”, the process of fabricating the covering layer involved depositing the Sol–gel not on a substrate, but rather directly on a typical solar cell.

## Results and discussion

### Crystal structure study by XRD

The pure ZnO film has the same wurtzite phase, the XRD of the Pure ZnO film has been discussed in details in previous work for the pure ZnO and the doped with Er ions one^9^; moreover, another previous work done by our team has depicted that the crystallite size of the pure sample is equal to 43 nm^13^.

**Fig. 1:**
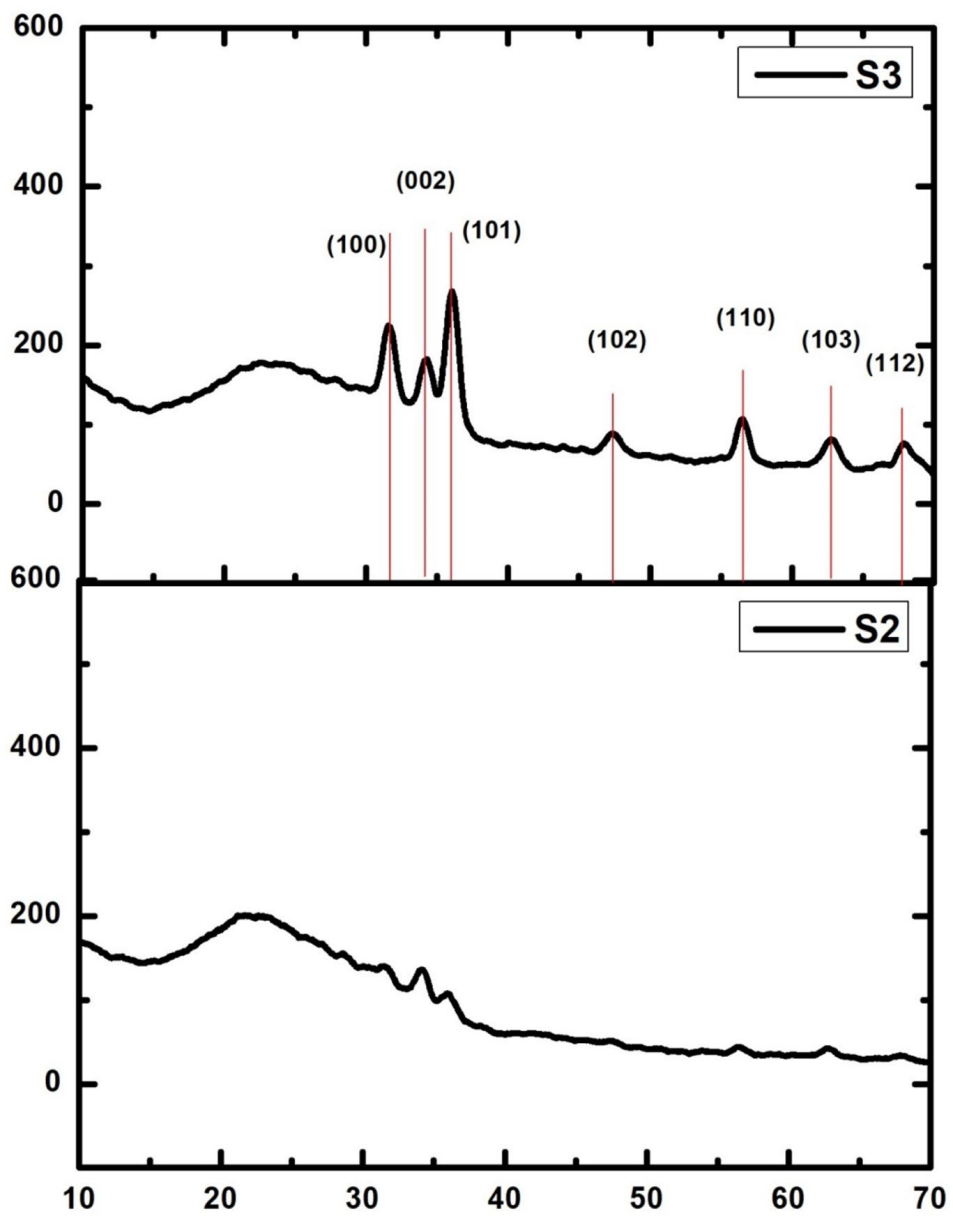
XRD spectra of S2 and S3, respectively**.**

Figure 1 illustrates the gathered diffraction spectra of the representative samples, S2 and S3, respectively, sintered at 500 °C. As can be seen, Sample S2 exhibits weak intensity peaks at diffraction angles 2θ equal to 31.68, 34.16, 36, 47.42, 56.56, 62.75, and 67.76°, in accordance with the ZnO wurtzite phase. The existence of weak diffraction peaks that match the ZnO orientation planes at (100), (002), (101), (102), (110), (103), and (212). Higher-intensity peaks emerged when the concentration of Yb^3+^ ions was increased, as in S3, resulting in noticeably enhanced crystallinity compared to S2. Peak locations in the two films under consideration are in good agreement with the hexagonal Wurtzite nano-structure ZnO reflections; all peaks match standard crystallographic data (ZnO: JCPDS 01–075-0576). Furthermore, as observed at 2θ° = 36°, the result demonstrated that ZnO exhibited a strong preferential growth orientation diffraction peak at the (101) plane, indicating that it is growing perpendicular to the substrate. In S2 and S3, respectively, the Wurtzite hexagonal phase with high c-axis orientations was observed. All obtained results are in agreement with the previously reported data^9–13^. Increasing the co-doping amount of Yb^3+^ ions, which enhances crystallinity and increases crystal size. Empirical observation has demonstrated that an increase in the percentage of Yb^3+^ ions causes a proportionate enlargement of the size of crystallites that range from 27.44 nm to 60.02 nm. This observation was made by detecting the crystal dimension values in the created samples. The fact that there are no further peaks associated with Er_2_O_3_ or Yb_2_O indicates that the dopants did not crystallize; instead, they filled the vacancies in the ZnO, i.e., the XRD data show that individual Zn^2+^ ions have been replaced individually by Er^3+^ and Yb^3+^ ions within the zinc oxide lattice. The rationale behind this is the notable difference in ionic radii between 0.74 Å for zinc, 0.868 Å for Yb^3^⁺, and 0.89 Å for Er^3^⁺. Moreover, when Yb^3^⁺ is introduced into Er-doped ZnO (Er-ZnO), the crystal size increases with increasing Yb^3^⁺ concentration. The presence of Yb^3^⁺ enhances ion diffusion and recrystallization kinetics, promoting the formation of larger crystals. High Yb^3^⁺ doping decreases grain boundary energy, thereby reducing nucleation sites for smaller grains and favoring the growth of larger grains instead^9,13^. Hence, assuming that rare earth elements disperse across several locations makes sense, as evidenced by the lower porosity of the background environment.

Peaks around 1.7, 2.2, and 3.6 correspond to the glass of the substrate, which was utilized as a substrate for the thin film^9^. In the same study, EDAX has been done and discussed the pure ZnO film and that doped with 1.5 mol.% of Er^3+^, which possesses near-stoichiometric composition. The ratio of Zn to O remains unchanged after the incorporation of Er^3+^. For the case of the pure ZnO film, the morphology appears to consist of interconnected spherical particles with pores present. These pores are filled lightly for the ZnO:1.5Er sample; this indicates that incorporating Yb^3+^ using the same preparation method will result in the exact stoichiometry.

### Bright field imaging

**Fig. 2:**
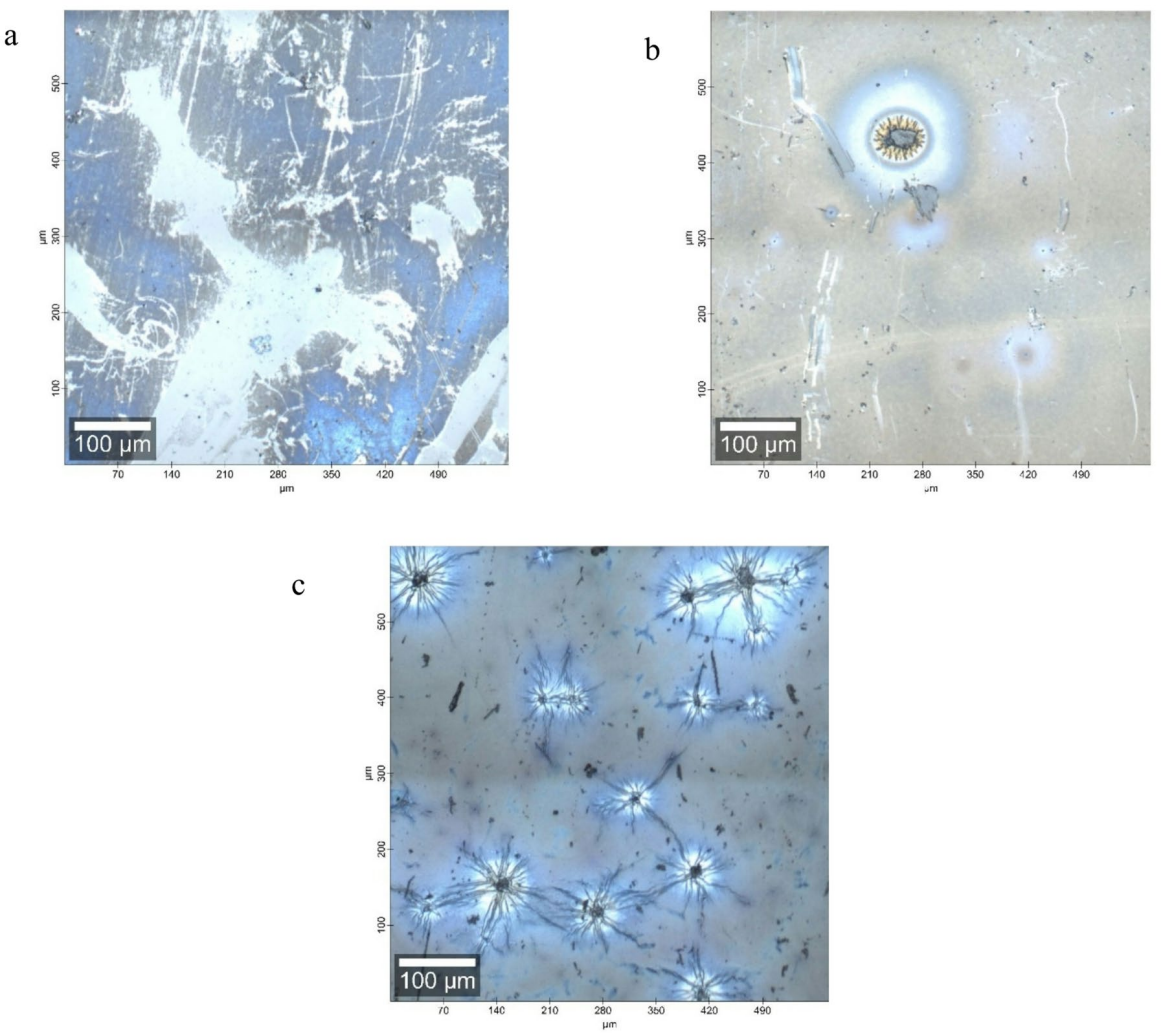
The optical images of the ZnO matrix co-doped with Er3+ and Yb3+; (**a**) S1, (**b**) S2, and (**c**) S3, respectively.

Figure 2 depicts the optical picture of all the samples. The optical images depict an aggregation of islands as the density of Yb^3+^ ions rises.

When comparing the photos with those in prior research that used phosphosilicate as a matrix instead of the ZnO matrix in the current study^44^, the impact of ZnO on enhancing the aggregation of the observed islands is apparent. The optical imaging aligns with the XRD spectra, as the XRD patterns clearly indicate an increase in crystallinity of ZnO with the addition of Yb3 + ions. This may be a reason for the formation of the gathered islands observed in the optical images.

### Spectrophotometry

#### Urbach energy and the optical E_g_

**Fig. 3:**
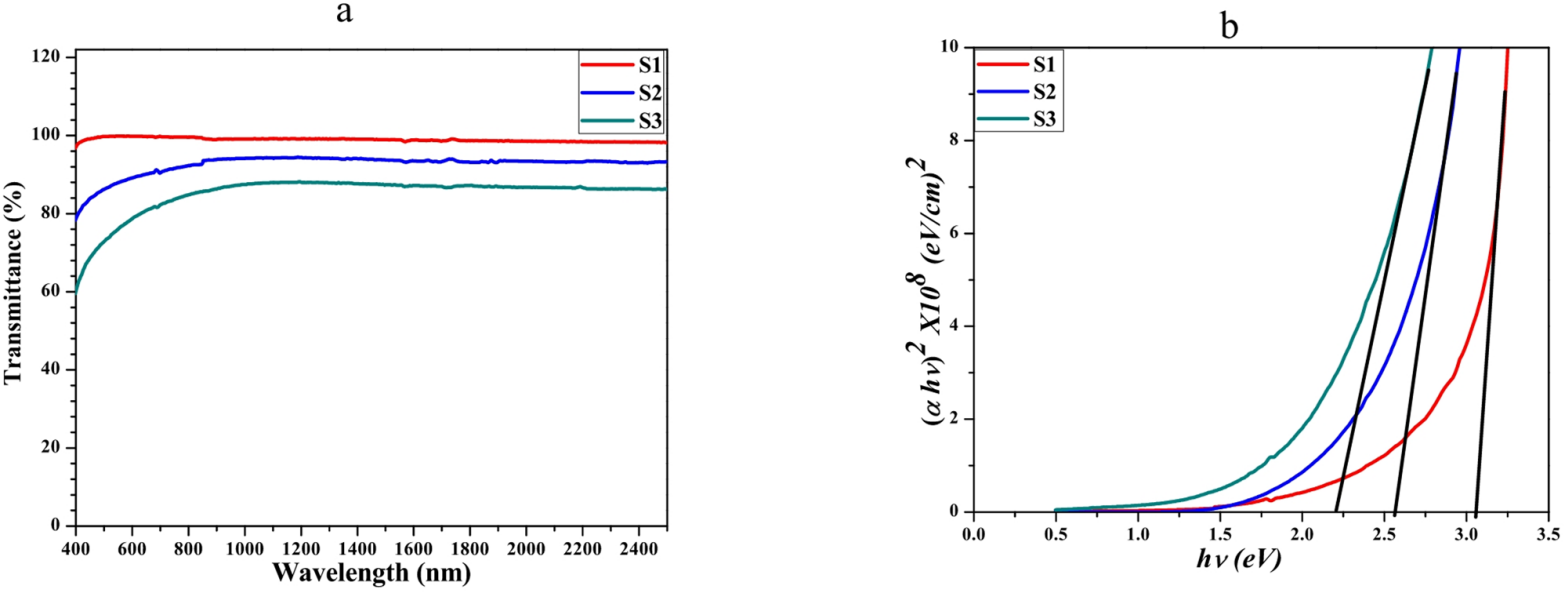
(**a**) Transmittance spectra, (**b**) The Tauc plot for the S1, S2, and S3 samples**.**

The three samples exhibit different amounts of optical transmittance, as shown in Fig. 3a. The S1, S2, and S3 exhibit exceptional transmittance across the visible and near-infrared (NIR) spectra. Moreover, S3 exhibits modest light transmission in the blue, green, red, and near-infrared wavelengths. This could be attributed to the absorption of oxygen deficits, characterized by defect levels that are marginally lower than the conduction band, consequently reducing the forbidden band^45,46^.

The optical Eg is an essential crucial quantity, considering the importance of electronic transitions. The Tauc plot, which is shown in Fig. 3b, was used to compute the optical Eg of the materials under investigation^47^. Samples S1, S2, and S3 had optical Eg of about 3.06, 2.56, and 2.2 eV, respectively. An increase in the mole percentage of Yb^3+^ ions from 0.5 to 1.5% results in a slight decrease in the Eg from 3.06 to 2.2 eV. A red shift near the absorption edge is observed with an increase in Yb3 + ion content. The band gap values are then decreased in a crystal structure by replacing Zn^2+^ ions with Yb^3+^ and Er^3+^ ions. This is since Yb, Er, and Zn have different ionic radii^48^. Moreover, the effect of photo-darkening, or the absorption edge red shift, signifies a narrowing of the films’ optical gap and may be caused by dopant hybridization with ZnO and Yb^3+^ ions concentration^9^. When dopants grow, these two factors may cause the valence and conduction bands to widen. Er-Yb increases the number of carriers in the ZnO’s conduction band. The energy interaction between free carriers causes Eg to drop when Yb^3+^ ion concentration rises, as Fig. 3b shows. Similar results have been reported in multiple studies using films based on ZnO, Co, Fe, or Mn produced by various methods^49–56^.

In addition, the absorption coefficient ($\alpha$) close to the main absorption threshold follows the empirically-based Urbach relation and has an exponential dependency on the input photon energy. The Urbach energy (E_u_) is a mathematical expression that quantifies the level of disorder in the film network:

2 $$\alpha = \alpha_{o} e^{{\frac{h\upsilon }{{E_{u} }}}}$$

**Fig. 4:**
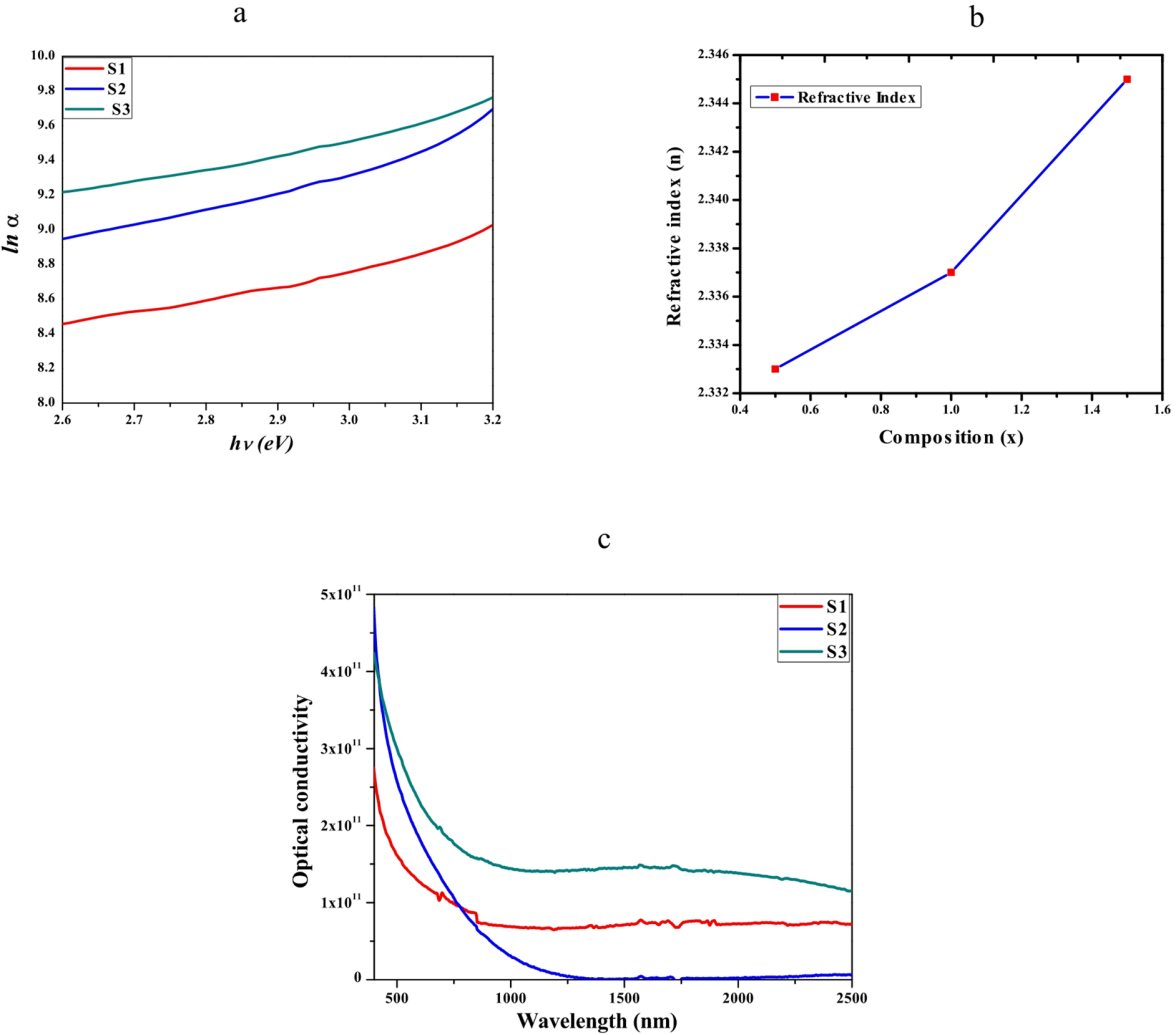
(**a**) Urach plot, (**b**) the refractive index against composition, (**c**) optical conductivity versus wavelength plots of the doped ZnO S1, S2, and S3 samples**.**

The constant α_o_ is temperature-independent and signifies the coordinates where the Urbach bundle converges. How wide the exponential absorption edge is one way to get E_u_. The E_u_ values for all samples were obtained by graphical analysis, as shown in Fig. 4a, where the photon energy (hυ) is plotted against the natural logarithm of the absorption coefficient (ln(α)) of the subjects under study. A direct proportionality between the Urbach energy and the concentration of Er-Yb is shown in Fig. 4a. A direct proportionality between the E_u_ and the concentration of Yb^3+^ ions is demonstrated by the data in Table 1. This relationship suggests that the Urbach energy grows in tandem with increases in photon energy and activated dopant Yb^3+^ concentration. The rise in Urbach energy can be attributed to the improvement in ZnO crystallinity, and the doping of Ytterbium leads to perturbation of the band structure through an increase in defect states. This could also be another explanation of the band gap contraction, as the band gap narrows due to the presence of localized states.

**Table 1:** The data for the samples S1, S2, and S3 that were studied include the following: optical density (OD), average optical electronegativity (χ), electron polarizability, direct Eg, Eu, refractive index (n), and so on, of S1, S2, and S3.

Sample number	Composition	E_g_ (eV)	E_u_ (eV)	n	Δχ	α_e_ at λ = 700 nm	OD at 3.5 eV
S1	0.5 Er−0.5 Yb	3.06	0.19	2.333	0.822	1.3 X 10^21^	0.278
S2	0.5 Er−1 Yb	2.57	0.24	2.337	0.691	1.2 X 10^21^	0.614
S3	0.5 Er−1.5 Yb	2.22	0.56	2.345	0.597	0.9 X 10^21^	1.559

#### Refractive index (n)

Because it plays a major part in determining which substances are used to build various optical applications, *n* is one of the distinctive and crucial properties of any material. Moreover, an important optical property, the refractive index, is firmly associated with the material’s local field and the electronic polarizability of its ions. The following equation expresses the direct correlation between the material’s optical energy gap and its refractive index:

3 $$\frac{{n^{2} - 1}}{{n^{2} + 2}} = 1 - \sqrt {\frac{{E_{g} }}{20}}$$

Table 1 demonstrates that the *n* rises when the doping concentrations of S1, S2, and S3 samples increase, reaching values of 2.333 for S1, 2.337 for S2, and 2.345 for S3. The relationship between the increase in dopant concentration and the rise in *n* can be explained by the higher *OD* of the Er-Yb dopants^56^, as seen in Fig. 4b. These findings indicate that the doping materials influence the refractive index values of the examined samples. Similar results have been observed in specific semiconductors and other materials^57^.

#### Optical conductivity (σ_opt_)

One measure of electric field strength produced by a substance’s induced current density is its optical conductivity, defined at a certain frequency as the strength of the field. Furthermore, the most straightforward way to learn about a substance’s optical behavior is to look at its *σ*_opt_. The calculation of *σ*_opt_ is performed using the following formula:

4 $$\sigma_{opt} = \frac{\alpha nc}{{4\pi }}$$

Figure 4c illustrates the graphical representation of the relationship between optical conductivity and wavelength for all the analyzed samples. In *σ*_opt_, it is widely recognized that α directly impacts the phenomenon and also the *n*. *σ*_opt_ displays a comparable trend to the absorption coefficient, suggesting that *σ*_opt_ decreases with increasing wavelength for the under-investigation samples. Furthermore, the presence of Er-Yb dopants at different concentrations substantially impacts the *σ*_opt_ of the samples. The conductivity improves as the dopant concentration increases, suggesting that these samples exhibit excellent photoresponse and can be utilized as photoconductors^58^. This discovery suggests that *σ*_opt_ of the current samples is influenced by the optical energy and the percentage of the Er-Yb dopant. Therefore, the current samples possess favorable characteristics that make them suitable for manufacturing thin-film solar cells; they are also highly suitable for many other optoelectronic devices^59^.

#### Skin depth (δ)

**Fig. 5:**
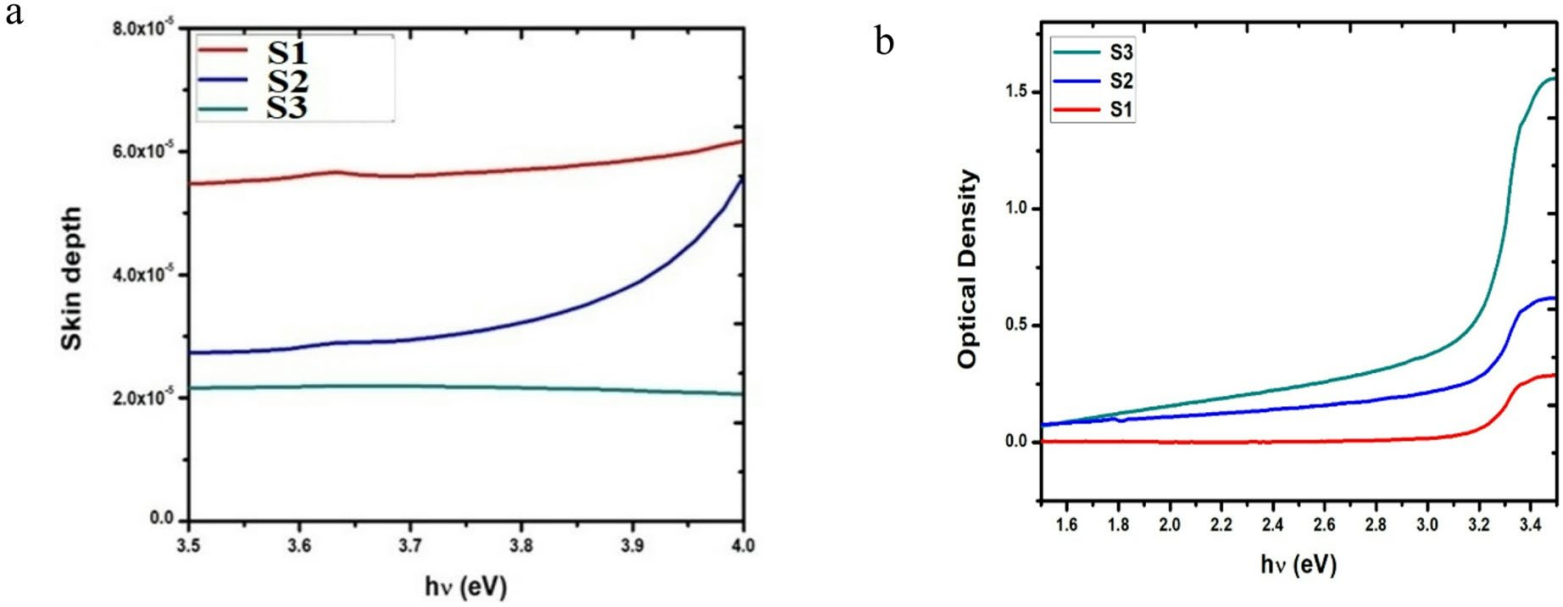
(**a**) The penetration depth, (**b**) The OD as a function of the photon energy for the co-doped ZnO samples S1, S2, and S3.

In microwave engineering, determining the skin depth is a crucial measurement. Furthermore, it also affects lower frequencies and is precisely defined as measuring the penetrability of electromagnetic radiation to a substance. If the thickness of these materials is sufficiently tiny, they will come across as transparent to the wavelength in question^60^. The skin depth is solely determined by the absorption coefficient (δ = 1/α). Figure 5a illustrates the relationship between the photon energy and **δ** for all doped samples. Increasing the Er–Yb doping concentration decreases the skin depth, suggesting that both photon energy and dopant concentration impact the skin depth. Given that the skin depth and dopant concentrations influence the absorption loss, establishing a relationship between skin depth and optical properties is essential.

#### Optical density (OD)

*OD* is the ability of such a material to transmit an amount of light through it. It measures the speed of light passing through a substance; it could be determined using the equation below:

5 $$Optical density \left( {OD} \right) = \alpha t$$

The term t represents the thickness of the three co-doped samples, namely S1, S2, and S3.

Figure 5b depicts the change in the *OD* as a relation with the photon energy just for the Er^3+^/Yb^3+^ co-doped samples; in this regime, it shows strong agreement with the transmittance spectra presented in Fig. 3a. Increasing the concentrations of Yb^3+^ dopant leads to a corresponding increase in the *OD* values.

From the figure, it is easily noticeable that the ZnO film doped with the highest concentration (1.5%) of Yb^3+^ (S3) sample has the deepest skin. The positive correlation between skin depth and the concentration of co-dopants enhances the optical characteristics, as absorption loss is a function of both. To observe the correlation between the increase in crystallite size from 27.44 nm to 60.02 nm for S2 and S3, respectively, in comparison with Eg and co-doping concentration, refer to Table 1.

### Laser raman spectroscopy

**Fig. 6:**
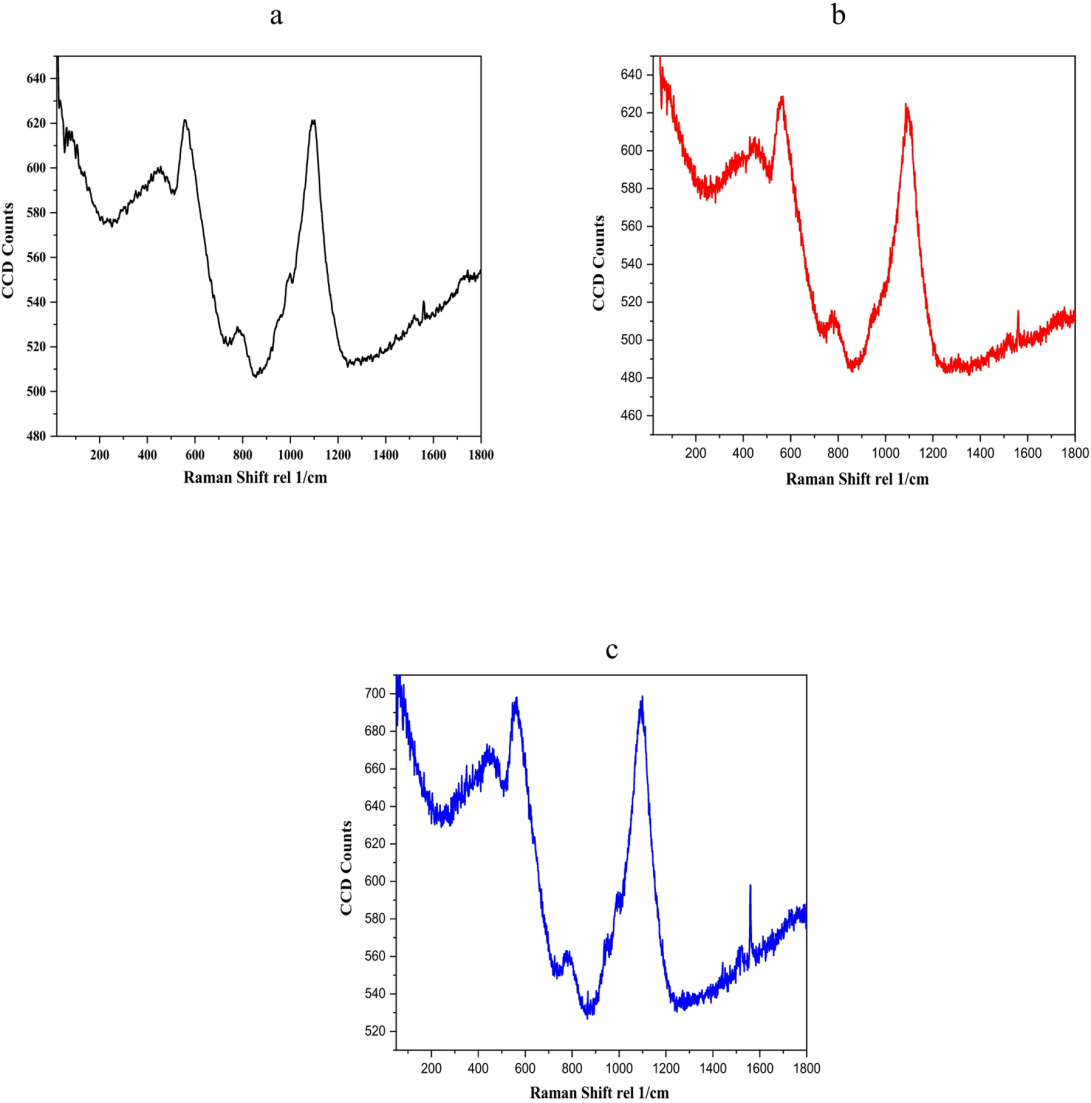
The Raman spectra of (**a**) S1, (**b**) S2, and (**c**) S3 thin films, respectively.

As presented in Fig. 2, the islands were clear and distributed in the S3 film, while you could find very few clear ones in the S2 film. In the S1 sample, it was tough to find such tiny ones. To understand the construction of the appeared islands, the Raman microscope was moved to one of them, and the 514 nm laser beam was directed to the point-making area to scan and average the spectra, as is clear in Fig. 6.

The ZnO Raman bands are shown in Fig. 6. The E2 (high) and second TO vibration modes of the Wurtzite structure correspond to the two most significant bands at 436 and 980 cm^-1^, respectively. It is well-known that Wurtzite has iron atoms within its hexagonal structure. However, these atoms did not show up in the Raman bands. According to the literature, the Wurtzite structure could still exist even if the iron ions migrate from the ZnO crystal^61^. In this case, it is most probable that the Er^3+^ and Yb^3+^ ions may be substituting for some dislocations of the iron atoms.

The electrically induced silent phonon (B1)^61^ may account for a further 580 cm^-1^ band. In all doped ZnO samples, especially those doped with 1.5% mol Yb^3+^, a Raman band at approximately 1072 cm^-1^ could be generated when the LO and TO optical phonons combine.

The 775 cm^-1^ band in the ZnO hexagonal Wurtzite Brillouin zone is a mix of the acoustic and optical phonons of the L-M line. This could explain the formation of ZnO crystals in connected islands, as observed in optical imaging^61^.

Both shoulders were determined to be temperature sensitive; the one at 950 cm^-1^ could be the second harmonic TO vibration, while the one at 1005 cm-1 could be a mixture of the TO and LO Raman modes. Both are often visible at ambient temperature and become even more so when cooled^62^.

According to the Raman spectra in Fig. 6, both shoulders were improved when the concentration of Yb^3+^ ions was increased to 1.5 mol% at room temperature. At the same time, both vibrations were related to the M, L, points, and M-L line in the ZnO hexagonal Wurtzite Brillion zone. This means an effect on the crystal vibrations is present, which could also explain the gathering of the mentioned islands in Fig. 2c.

As per the energy-time uncertainty relation^63^, the phonon lifetime may be determined from the Raman spectra by using the FWHM in cm^-1^ (Γ) as:

6 $$\tau = \frac{1}{2\pi c\Gamma }$$

The phonon decay time is usually controlled by one of the following: either the anharmonicity of the phonon decay to more than one phonon or the perturbation of the translational symmetry of the crystal as a result of any ultra-fine additive or any crystal defects. Our situation falls under the second category: the crystal’s translational symmetry disruption caused by very minute additive or crystal imperfections. Consequently, Yb^3+^ ions introduced into a ZnO crystal alter phonon decay, generating a secondary additional optical and acoustic phonons. Studying the influence of increasing the Yb^3+^ ion contents on the ZnO crystal, the FWHM of the Raman band at 775 cm^-1^ was selected as a monotonic vibrational mode, as previously stated. For each set of observed Raman spectra, the phonon decay time is shown in Table 2 as the FWHM of the 775 cm^-1^ band.

**Table 2:** The FHWM of Raman bands at 580 cm^-1^ as standard ZnO bands.

Sample	FHWM (cm^−1^)	Decay time (ps)
S1	36	14.8
S2	35	15
S3	33	16.1

As shown in Fig. 6, the bandwidth in both S1 and S2 is approximately similar; consequently, the decay time appears to be roughly the same. However, the increase in Yb3 + concentration to 1.5 mol.% broadens the Raman band, thereby increasing the phonon decay time. The phonon decay time is a strong indicator of the modes of vibration in the ZnO crystal lattice and provides evidence about the effect of Yb^3+^ ions on producing new generations of optical and acoustic phonons.

The presence of phonons modulates the dielectric function, which can significantly increase the photon absorption through electron–phonon interaction, thereby improving solar cell efficiency.

### The photoluminescence up-down conversion (PLUDC)

The fundamental process of PLUDC is believed to involve energy transfer (ET) occurring between the excited Yb^3+^ and Er^3+^ ions. When Ytterbium ions are employed as a sensitizer to activate the energy states of the Erbium ions in a ZnO host material, the states of the Erbium ions undergo such rearrangement. Yb^3+^ ions serve as a sensitizer for up-down conversion (UDC) fluorescence radiation from Erbium ions’ states, particularly when the probability of the absorption process is negligible. Ytterbium ions that compete with the transition of Er3+ ions in a proper spectrum enable a more efficient electron transfer from Ytterbium ions to Erbium ions, resulting in fluorescence radiation from Erbium. This method works well when using Yb^3+^ ions at a concentration of 1.5 mol%. A wider absorption cross-section band is predicted for Yb^3+^ ions. The ^2^F_7/2_ → ^2^F_5/2_ transition of Ytterbium ions is highly compatible with these transitions, facilitating efficient energy transfer (ET) from Yb3 + to neighboring ions, making them a promising up-conversion down-conversion (UCD) sensitizer^47,64^.

**Fig. 7:**
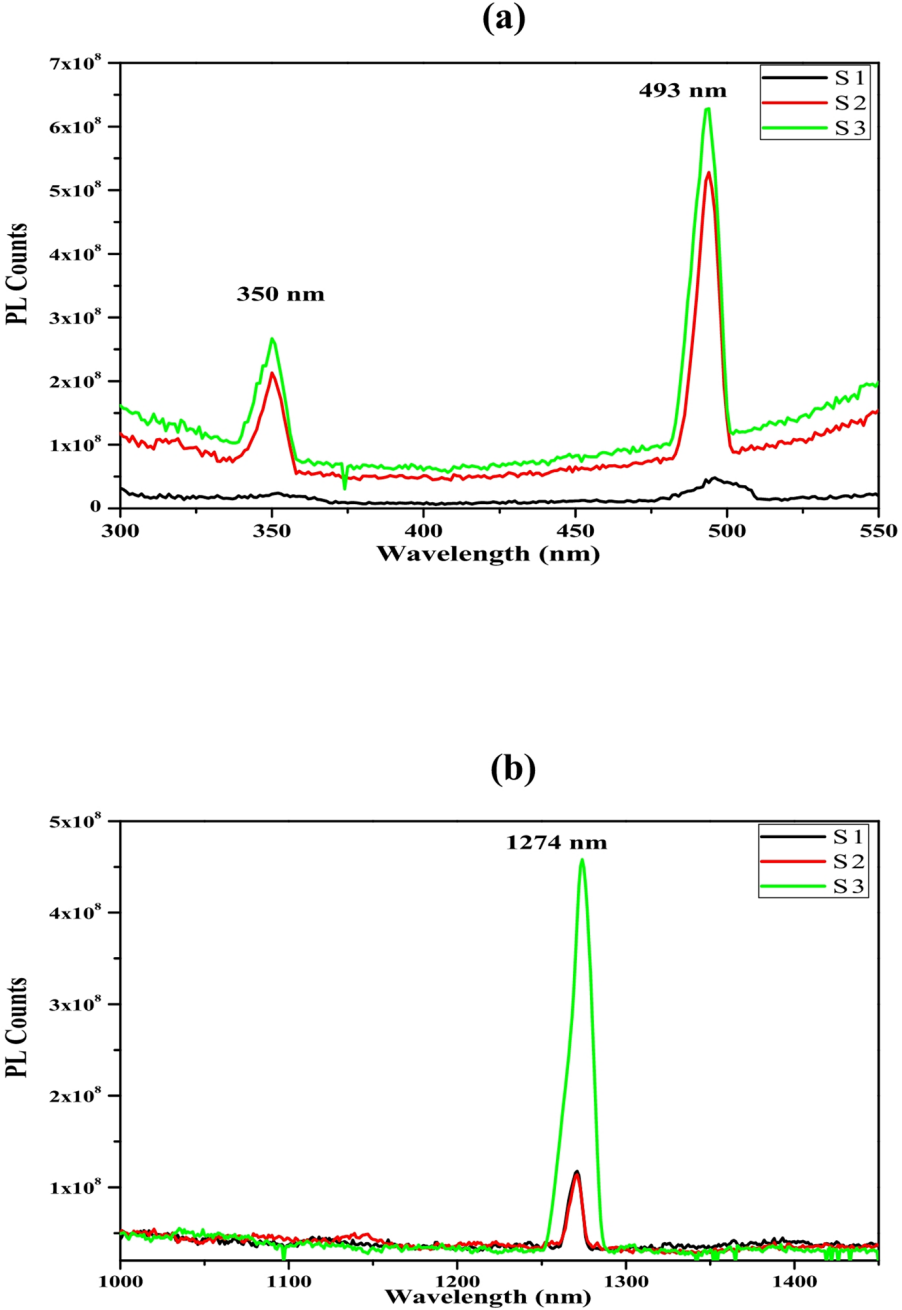
The PL of S1, S2, and S3, respectively. Excitation with a 635 nm laser for (**a**) UC and (**b**) DC.

Using a 635 nm laser to stimulate the nano-structured thin films S1, S2, and S3, we can see how the PL emission of these films changes as the concentration of Yb^3+^ ions increases. As its primary focus, this research investigates the impact of the UDC phenomenon on solar cells. Figure 7a,b show the documented findings. It has been noted that a stronger emission at greater Yb^3+^ ion contents, notably at 1.5 mol %, is produced by raising the concentration of Yb^3+^ ions in the up-conversion process. Further, there is a slight peak shift to lower wavelength from 352 to 350 nm at this Yb^3+^ ions concentration. Another shift occurs from 497 to 493 nm in the strong green peak emission at the two higher ytterbium ion concentrations, Fig. 7a. The mentioned shifts could be taken into consideration, especially since the spectral resolution of the FS5 spectrofluorometer is 0.5 nm.

The effect due to the increase in the sensitizer Yb^3+^ ion content shows different behavior for the down-conversion process, as presented in Fig. 7 b. The shift occurs exclusively at 1.5 mol% and not at the other two lower concentrations, in addition to the obvious augmentation of the amount of emitted photons for certain PL bands of the Er^3+^ ion. Moreover, the broadening occurs very strongly at 1.5 mol. % of Yb^3+^. This could explain the effect of 1.5% mol, as noticed in the Raman measurements, where the phonon time decay is approximately the same for both 0.5% and 1.0% mol, then increases when the concentration reaches 1.5% mol. %.

Such explanation for the broadening and shifting is that the increase of Yb ion to 1.5% affects the crystal structure and generates secondary phonons, so as the crystal structure affected it should be expected some changes in the electronic level system which is noticed in the PL spectra by broadening which means that the electrons take longer time from the excitation to relax to the ground state. Additionally, the generated photons may interact with the excited photons by pumping them into higher rotational states within the main energy level, resulting in a shift in the wavelength of the emitted photons.

The thin films that were discussed before showed a robust green PL emission at 497 nm when stimulated at 635 nm. The ^4^F_3/2_ and ^4^F_5/2_ sublevels of the Er^3+^ ions were thought to have caused this emission via their intra-4F transitions to the ^4^I_15/2_ sublevel. This ^2^G_7/2_ → ^4^I_15/2_ transition of the Er^3+^ ions was linked to a less strong UV peak PL emission at 350 nm, as seen in Fig. 7a. Figure 7b shows that during the down-conversion process, there was a noticeable emission band at 1274 nm, which may have been caused by the following intra 4F transition: ^4^I_11/2_ → ^4^I_15/2_ of Er^3+^ ions.

These two behaviors reflected the Up-down conversion harvesting solar cell process presence in the prepared samples, in which Er^3+^ ions absorb low-energy photons at 635 nm. Figure 7a,b show the resulting photon emissions, which were 350 and 498 nm for greater energy and 1274 nm for lower energy, as described in reference^64^.

During the PLUDC emission process, several phenomena were exhibited, such as ET and excited-state absorption (ESA). Additionally, PLUDC offers ground-state absorption (GSA) and non-radioactive relaxation techniques. A single-photon GSA process excites both Erbium and Ytterbium ions from their respective ground states ^2^F_7/2_ and ^4^I_15/2_ to the ^2^F_5/2_ and ^4^I_11/2_ sub-levels. The energy of Ytterbium ions is delivered to the ^4^I_11/2_ level of Erbium ions from the ^2^F_5/2_ level.

The ^2^P_3/2_, ^4^F_5/2_, and ^4^I_11/2_ sub-levels are filled with Erbium ions by the ESA process. The excited Erbium ions occupy the ^2^P_3/2_, ^4^F_5/2_, and ^4^I_11/2_ sub-levels via phonon relaxation. After this, it relaxes to non-radioactive levels at ^2^G_7/2_, ^4^F_7/2_, and ^4^I_13/2_.

The radioactive transition emits photons with wavelengths of 350 nm (UV), 497 nm (green), and 1273 nm (IR). The emissions may be attributed to specific transitions within the 4I energy level, including the ^2^G_7/2_, ^4^F_7/2_, and ^4^I_13/2_ → ^4^I_15/2_ transitions. This validates the existence of PLUDC. The addition of Yb^3+^ ions to ZnO films has increased the visibility of PLUDC emission bands. This enhancement may be due to a more effective energy transfer from the 2F5/2 of the Ytterbium ions to the 4I11/2 of the Erbium ions, or it may be caused by preventing Er2O3 clustering within the films. Ions of Er^3+^ and Yb^3+^ that had relaxed to their ground states were excited by a 635 nm laser, and radiation of ultraviolet, visible, and infrared wavelengths was emitted. The Ytterbium emission underwent cross-relaxation, causing the electrons in the ^4^I_11/2_ state of Erbium to transition to a higher energy state, namely the ^4^F_7/2_ state of Yb^3+^ ions.

One can conclude that to enhance the photoluminescence efficiency of ZnO doped with Er3+, a possible approach is to introduce Yb3+ ions through co-doping. The presence of Yb^3+^ ions in Er^3+^ doped nano-structure materials is widely recognized to have a substantial sensitizing impact. It can be inferred that the emission caused a significant green PL emission at 497 nm, which may be attributed to ^4^F_7/2_→^4^I_15/2_ intra 4F transitions of the Er^3+^ ions. Additionally, a less intense blue peak PL emission at 350 nm corresponds to another intra-4F transition of Er^3+^ ions, specifically the 2G7/2•4I15/2 transition, known as up-conversion.

Alternatively, the down-conversion process was feasible by a different inter-4F transition involving Er^3+^ ions, namely from ^4^I_13/2_ to ^4^I_15/2_^47,63,65^.

### Effect of applying S3 sample upon solar cell

**Fig. 8:**
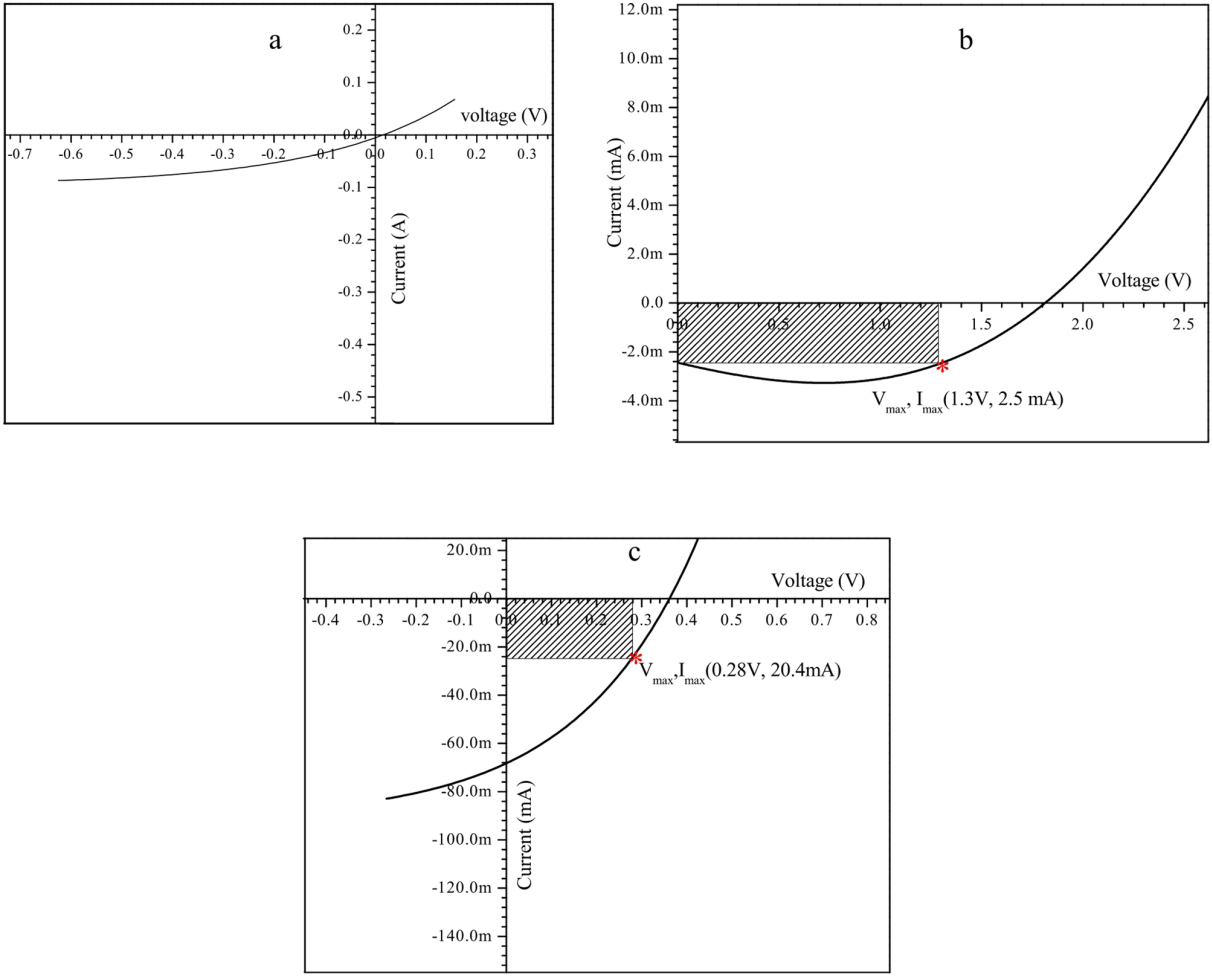
The typical solar cell’s I-V graph, (**a**) after S3 deposition in the dark, (**b**) before, (**c**) after S3 deposition, both (**b**, **c**) at room temperature, were exposed to a white light source having a power density of 32 Wm^−2^.

The voltage-current (I-V) curve of a control sample of conventional solar cells was examined prior to S3 deposition, as shown in Fig. 8a. An illumination source consisting of white light with a power density of 32 Wm^-2^ was used to experiment with the S3 sample deposition on the front of the silicon solar cell. The potential was recorded at 1.3 volts, while the current peaked at 2.5 amps. The tested solar cell has an approximate open-circuit voltage (Voc) of 1.8 V and a short-circuit current (Isc) of 2.2 mA. Although the generated samples have been verified as materials for Photoluminescence Up-Down shifting harvesting higher efficiency solar cells (PLUDCHSC). Using them in solar cells could increase the amount of solar energy that is harvested. According to Section “Covering the solar cell”, the sample doped with 0.5 mol% Er and 1.5 mol% Yb (S3) was selected in this regime. For example, the ZnO thin film S3 deposited on the surface of the Si solar cell can be examined, as shown in Fig. 8a. We began by reviewing the dark state stage. Figure 8a lacks current gain, but the curve indicates that a solar cell-capable connection is forming. Then, when the prepared samples have been confirmed as Photoluminance Up-Down shifting harvesting higher efficiency solar cell (PLUDCHSC) materials, applying them to solar cells may enhance their harvesting of a broader range of solar energy. The sample doped with 1.5 mol% Yb (S3) was chosen for this purpose, as described in Section “Covering the solar cell”. With a doping ratio of 0.5%:1.5% Er^3+^: Yb^3+^ ions^47^, for instance, the ZnO thin film co-doped with Er^3+^/Yb^3+^ ions deposited on the surface of the Si solar cell may be studied. We began by examining the dark state stage, as shown in Fig. 8a. Although the lack of current gain is evident, the curve indicates the formation of a solar cell-capable connection. After depositing S3 sample to the front of silicon solar cell-based devices, the higher efficiency of collecting solar cell energy increased, rising from 10.5% up to 13.1%, respectively with the benefit of very simple and in-expensive processing.

Figure 8b shows the diagram of the I-V graph for the conventional solar cell subjected to light at 32 Wm^-2^ following S3 deposition. Following the addition of sample S3 to the traditional solar cell, it was observed that a greater current flowed through the electrodes under identical lighting conditions; the highest voltage and current measurements were 0.28 V and 20.4 mA, respectively, as shown in Fig. 8b.

A produced solar cell’s efficiency is a crucial metric to consider. We might use this equation to figure out the efficiency of the solar cell (ɳ):

7 $$\eta = \frac{{Ir_{out} }}{{Ir_{in} }} \times 100{\text{\% }}$$

where, Ir_out_ and Ir_in_ represent the solar cell’s electrical power per unit area and the incident light intensity, respectively. The corresponding maximum current (I_m_), which produces the maximum power (P_m_) due to maximum potential difference (V_m_), could be calculated as:

8 $$I_{m} = { }{\raise0.7ex\hbox{${P_{m} }$} \!\mathord{\left/ {\vphantom {{P_{m} } {V_{m} }}}\right.\kern-0pt} \!\lower0.7ex\hbox{${V_{m} }$}} = 0.0{42}\,{\text{A}}$$

When calculating the solar cell’s efficiency, the fill factor (FF) is a crucial electrical parameter. There is a direct correlation between the two variables; therefore, raising FF improves the efficiency of power conversion in solar cells. FF could be calculated utilizing the following relation:

9 $$FF = \frac{{V_{m} I_{m} }}{{V_{oc} I_{sc} }}$$

Figure 8’s results, when combined with Eqs. 10 and 11, make it easy to see how depositing thin films of ZnO co-doped with Er3 + /Yb3 + nanostructures affects the solar cell’s efficiency. Table 3 displays the calculated solar cell properties.

**Table 3:** The solar cell properties both before and after the deposition of the S3 sample.

Before the S3 sample deposition	After S3 sample deposition
(I_m_) = 0.0024 A	(I_m_) = 0.068 A
V_m_ = 1.318 V	V_m_ = 0.36 V
FF = 0.728	FF = 0.327
${\upeta }$ = 10.544%	${\upeta }$ = 13.333%

The data shown in Table 3 demonstrates that the utilization of the S3 sample on the surface of the silicon solar cell leads to a significant enhancement in the solar cell’s ability to convert light into electricity, rising from 10.544 percent to 13.333 percent. The rise in short circuit current may be ascribed to the presence of (PLUDC); this originates from the accumulation of ZnO that has Er^3+^/Yb^3+^ as impurities. The third sample, S3, exhibits the most pronounced influence, evidenced by its highest PL intensity peaks. Considering this, S3 could potentially enhance the efficacy of solar cells in PLUDCHSC applications. The emission band stated earlier is situated in the visible spectrum and falls within the absorption range of silicon solar cells^65,66^.

## Conclusion

Co-doping ZnO NS thin films with constant concentrations of Er^3+^ ions and different levels of quantity of Yb^3+^ ions at (0.5, 1.0, and 1.5 mol. %) via SSSCSGM preparation, resulting in exceptionally transparent films. The sintering aids were engaged at a temperature of 500 °C. Aiming to apply coating layers of the ZnO films co-doped with 0.5 and 1.5 mol. % of Er and Yb ions, (S3) sample, which has the largest (HPLUDCSC) band emissions, on the front side surface of the silicon solar cell, causing a significant enhancement in the solar cell’s ability to convert light into electricity, rising from 10.544 percent to 13.333 %. Specifically, it was proven that the (PL) intensity was affected by the Yb^3+^ ions content, which acts as a sensitizer to Er^3+^ ions. The up-shifting and down-shifting areas showed increased intensity under 635 nm laser line excitation, with the greatest increase seen for 1.5 mol. % of Yb^3+^ ions, S3. The (UC) process of another intra-4F transition of Er^3+^ ions at ^2^G_7/2_→^4^I_15/2_ led to a less strong ultraviolet PL emission at 350 nm. Furthermore, at 1274 nm, the ^4^I_11/2_→^4^I_15/2_ transition, due to another intra-4F transition for (DC), was observed.

For this purpose, various spectroscopic techniques, such as XRD, spectrophotometry, and laser spectroscopy, have been employed to examine the photo-physical characteristics, construction, morphology, and optical properties. The XRD and Raman spectra confirmed that the films show a Wurtzite crystal structure. XRD analysis revealed a positive correlation between the peak intensities and the concentration of Yb^3+^ ions, indicating an increase in the degree of crystallinity. The Raman micro-spectroscopy can identify the Brillouin zones of crystalline films, providing further structural confirmation of the produced samples. The transmission spectra of doped ZnO samples S1, S2, and S3 exhibited optical transparency in the visible range. Through the analysis of the absorption spectra, we successfully determined the optical bandgap (E_g_) and its corresponding characteristics. Estimations were made for the OD, skin depth, E_g_, and E_u_. Furthermore, the anticipated E_u_ exhibits a higher magnitude for all samples as the quantities of Yb^3+^ dopants are augmented.

The results show that promising photoluminescence up-down shifting harvesting (PLUDCH) for higher efficiency solar cell applications may benefit greatly from ZnO NS co-doped with 0.5 mol. % Er^3+^ and 1.5 mol. % Yb^3+^ ions, (S3) prepared sample.

## Data Availability

All data generated or analysed during this study are included in this published article.
